# Supplementary material for: Genotypic and Epidemiologic Profiles of Giardia duodenalis in Four Brazilian Biogeographic Regions
Source: Microorganisms. 2022 Apr 30;10(5):940. doi: 10.3390/microorganisms10050940 (PMC9142931; doi:10.3390/microorganisms10050940)
Supplement: Supplementary file 1 [file microorganisms-10-00940-s001.zip › microorganisms-1591385-supplementary.pdf]

microorganisms

MDPI

Supplementary Materials

Table S1. *Giardia duodenalis* reference strains used in this study.

Accession Number	Isolate	Assemblage / Genotype	Host	Country	Content	Year	Reference
EU769206	Swecat171	A	cat	Sweden	Europe	2011	[1]
EU621373	JC002	A	deer	Poland	Europe	2008	[2]
EU769205	Swecat078	A	cat	Sweden	Europe	2008	[1]
GU396696	H3-001	A	human	Poland	Europe	2016	[3]
HM165227	Sweh040	A	human	Sweden	Europe	2016	[4]
KF922979	HC11	A	human	Brazil [a]	South America	2014	[5]
KF922983	HC29	A	human	Brazil [a]	South America	2014	[5]
KF922992	HC44	A	human	Brazil [a]	South America	2014	[5]
KF923000	DC01	A	dog	Brazil [a]	South America	2014	[5]
KM190682	VANC/85/UBC/2	A	human	Canada	North America	1985	[6]
KY612245	AN1.5	A	human	Brazil [b]	South America	2014	[7]
MN629930	Z1	A	dog	Iraq	Asia	2020	[8]
XM001705373	WB C6	A	culture	USA	North America	2007	[9]
GQ329671	Sweh166	A	human	Sweden	Europe	2011	[4]
EU014384	Be2	A1	beaver	Canada	North America	2011	[10]
HQ179591	46c2	A2	human	Australia	Oceania	2011	[11]
AY072724	ISSGF7	A3	human	Italy	Europe	2002	[12]
DQ090542	STS-U	A3	human	Norway	Europe	2004	[13]
DQ116612	CBHRG9	A3	wastewater	Mexico	North America	2006	[14]
EU014381	AB	A3	human	Peru	South America	1985	[15]
FJ560577	Lim2	A3	human	France	Europe	1998	[16]
FJ971408	GLT1	A3	human	Thailand	Asia	2007	[17]
FN386484	I231104	A3	wastewater	Spain	Europe	2004	[18]
KU504738	S42	A3	human	Brazil [c]	South America	2011	[19]
DQ090523	BG-Ber2	B	human	Norway	Europe	2005	[13]
AB480877	PalH9	B	human	Palestine	Asia	2006	[20]
AB618785	GH-202	B	human	Japan	Asia	2010	[21]
FJ971440	GLT2	B	human	Thailand	Asia	2007	[17]
FJ971461	GL518	B	human	Thailand	Asia	2010	[17]
FJ971482	GL121	B	human	Thailand	Asia	2007	[17]
KF922985	HC32	B	human	Brazil [a]	South America	2014	[5]
KF922993	HC45	B	human	Brazil [a]	South America	2014	[5]
KP687755	VANC/90/UBC/44	B	wastewater	Canada	North America	1990	[6]
KP687756	"VANC/90/UBC/54	B	beaver	Canada	North America	1990	[6]
KU504702	S3C1	B	human	Brazil [c]	South America	2011	[19]
KU504704	S3C3	B	human	Brazil [c]	South America	2011	[19]
KU504707	S8	B	human	Brazil [c]	South America	2011	[19]
KU504708	S9	B	human	Brazil [c]	South America	2011	[19]
KU504709	S10C3	B	human	Brazil [c]	South America	2011	[19]
KU504712	S11C1	B	human	Brazil [c]	South America	2011	[19]
KU504713	S11C2	B	human	Brazil [c]	South America	2011	[19]
KU504714	S11C3	B	human	Brazil [c]	South America	2011	[19]
KU504715	S13C2	B	human	Brazil [c]	South America	2011	[19]
KU504720	S15	B	human	Brazil [c]	South America	2011	[19]
KU504722	S16C2	B	human	Brazil [c]	South America	2011	[19]
KU504723	S16C4	B	human	Brazil [c]	South America	2011	[19]
KU504731	S24C2	B	human	Brazil [c]	South America	2011	[19]
KU504732	S24C3	B	human	Brazil [c]	South America	2011	[19]
KY612242	S8C5	B	human	Brazil [c]	South America	2011	[19]
LC508615	K6 clone1	B	human	Kenya	Africa	2013	[22]
MT542771	5	B	human	Brazil [c]	South America	2021	[23]
AY072725	Nij5	B1	human	The Netherlands	Europe	2002	[24]
AY072726	LD18	B2	human	Belgium	Europe	2002	[24]
AY072727	BAH8	B3	human	Australia	Oceania	1999	[12]
JF422719	BRAdogD15	C	dog	Brazil [a]	South America	2009	[25]

KF923019	VET01	D	dog	Brazil [a]	South America	2014	[5]
AY072729	P15	E	pig	Czech Republic	Europe	1996	[12]
AY647264	A101	F	cat	Italy	Europe	2004	[26]

[a] Atlantic Forest biome; [b] Cerrado biome; [c] Amazon biome.

Table S2. Molecular diversity indexes of *Giardia duodenalis* based on β-giardin locus (592 bp, n=106).

Assemblage	Region (N)	Statistics					
		H ± SD	Nº of haplotypes	Nº of polymorphic sites	Nº of substitutions	Nº of transitions	Nº of transversions
Assemblage A	South America (31)	0.879 ± 0.037	14	38	39	21	18
	North America (4)	0.666 ±0.204	2	4	4	4	0
	Europe (10)	0.822 ± 0.096	5	15	15	15	0
	Asia (2)	1.000 ± 0.500	2	4	4	4	0
	All continents (49)	0.854 ± 0.029	16	50	50	33	18
	All Amazon biome (5)	0.800 ± 0.164	3	8	8	6	2
	**Amazon biome* (4)**	0.833 ± 0.222	3	8	8	6	2
	All Cerrado biome (8)	0.964 ± 0.077	7	30	31	15	16
	**Cerrado biome* (7)**	1.000 ± 0.076	7	30	31	15	16
	**Caatinga biome* (5)**	0.700 ± 0.218	3	2	2	2	0
	All Atlantic Forest biome (13)	0.859 ± 0.063	6	6	6	6	0
	**Atlantic Forest biome* (9)**	0.750 ± 0.112	4	4	4	4	0
	Atlantic Forest biome (4)	0.833 ± 0.222	3	2	2	2	0
	All Brazil (31)	0.879 ± 0.037	14	38	39	21	18
Assemblage B	South America (41)	0.918 ± 0.033	23	36	36	31	6
	North America (2)	1.000 ± 0.500	2	3	3	3	0
	Europe (3)	1.000 ± 0.272	3	7	7	7	0
	Asia (5)	1.000 ± 0.126	5	5	5	5	0
	All continents (51)	0.921 ± 0.028	28	37	37	32	6
	All Amazon biome (37)	0.899 ± 0.039	19	24	24	23	1
	**Amazon biome* (21)**	0.757 ± 0.086	7	8	8	7	1
	Amazon biome (16)	0.991 ± 0.025	15	21	21	21	0
	All Atlantic Forest biome (3)	1.000 ± 0.272	3	10	10	7	3
	Atlantic Forest (2)	1.000 ± 0.500	2	5	5	4	1
	All Brazil (41)	0.918 ± 0.033	23	36	36	31	6
**ALL (106) ****		0.951 ± 0.009	50	125	147	105	42

H ± SD: gene diversity ± standard deviation. (bp): base pair. * Sequences obtained in this study **ALL: included assemblages A, B, C, D, E and F. Further details of reference strains can be found in Supplementary Table S1. Only groups with more than 1 sequence are shown.

References

1. Lebbad M, Mattsson JG, Christensson B, Ljungström B, Backhans A, Andersson JO, et al. From mouse to moose: multilocus genotyping of *Giardia* isolates from various animal species. Vet Parasitol. 2010;168(3-4):231-9. doi: 10.1016/j.vetpar.2009.11.003. PMID:19969422.
2. Solarczyk P, Majewska AC, Moskwa B, Cabaj W, Dabert M, Nowosad P. Multilocus genotyping of *Giardia duodenalis* isolates from red deer (Cervus elaphus) and roe deer (Capreolus capreolus) from Poland. Folia Parasitol (Praha). 2012;59(3):237-40. doi: 10.14411/fp.2012.032 PMID: 23136805.
3. Solarczyk P, Werner A, Majewska AC. Genotypowanie izolatów *Giardia duodenalis* uzyskanych od ludzi w zachodnio-centralnej Polsce [Genotype analysis of *Giardia duodenalis* isolates obtained from humans in west-central Poland]. Wiad Parazytol. 2010;56(2):171-7. PMID: 20707303.
4. Lebbad M, Petersson I, Karlsson L, Botero-Kleiven S, Andersson JO, Svenungsson B, et al. Multilocus genotyping of human *Giardia* isolates suggests limited zoonotic transmission and association between assemblage B and flatulence in children. PLoS Negl Trop Dis. 2011;5(8):e1262. doi: 10.1371/journal.pntd.0001262 . PMID: 21829745.
5. Durigan M, Abreu AG, Zucchi MI, Franco RMB, de Souza AP. Genetic Diversity of *Giardia duodenalis*: Multilocus Genotyping Reveals Zoonotic Potential between Clinical and Environmental Sources in a Metropolitan Region of Brazil. PLoS ONE. 2014 9(12): e115489. doi:10.1371/ journal.pone.0115489.
6. Prystajecky N, Tsui CK, Hsiao WW, Uyaguari-Diaz MI, Ho J, Tang P, et al. *Giardia* spp. Are Commonly Found in Mixed Assemblages in Surface Water, as Revealed by Molecular and Whole-Genome Characterization. Appl Environ Microbiol. 2015;81(14):4827-34. doi: 10.1128/AEM.00524-15 PMID: 25956776.
7. Nunes BC, Calegar DA, Pavan MG, Jaeger LH, Monteiro KJL, Dos Reis ERC, et al. Genetic diversity of *Giardia duodenalis* circulating in three Brazilian biomes. Infect Genet Evol. 2018;59:107-112. doi: 10.1016/j.meegid.2018.02.001 PMID: 29410226.

8. Hassan,Z.I. Unpublished. Avaible in {HYPERLINK "https://www.ncbi.nlm.nih.gov/nuccore/MN629930".
9. Morrison HG, McArthur AG, Gillin FD, Aley SB, Adam RD, Olsen GJ, et al. Genomic minimalism in the early diverging intestinal parasite *Giardia lamblia*. Science. 2007;317(5846):1921-6. doi: 10.1126/science.114383 PMID: 17901334.
10. Teodorovic S, Braverman JM, Elmendorf HG. Unusually low levels of genetic variation among Giardia lamblia isolates. Eukaryot Cell. 2007;6(8):1421-30. doi: 10.1128/EC.00138-07. PMID: 17557879; PMCID: PMC1951139.
11. Wielinga C, Ryan U, Andrew Thompson RC, Monis P. Multi-locus analysis of *Giardia duodenalis* intra-Assemblage B substitution patterns in cloned culture isolates suggests sub-Assemblage B analyses will require multi-locus genotyping with conserved and variable genes. Int J Parasitol. 2011;41(5):495-503. doi: 10.1016/j.ijpara.2010.11.007 PMID: 21176781.
12. Cacciò SM, De Giacomo M, Pozio E. Sequence analysis of the beta-giardin gene and development of a polymerase chain reaction-restriction fragment length polymorphism assay to genotype *Giardia duodenalis* cysts from human faecal samples. Int J Parasitol. 2002;32(8):1023-30. doi: 10.1016/s0020-7519(02)00068-1 PMID: 12076631.
13. Robertson LJ, Hermansen L, Gjerde BK, Strand E, Alvsvåg JO, Langeland N. Application of genotyping during an extensive outbreak of waterborne giardiasis in Bergen, Norway, during autumn and winter 2004. Appl Environ Microbiol. 2006;72(3):2212-7. doi: 10.1128/AEM.72.3.2212-2217.2006 PMID: 16517674.
14. Di Giovanni GD, Betancourt WQ, Hernandez J, Assadian NW, Flores Margez JP, Lopez EJ. Investigation of potential zooanthroponotic transmission of cryptosporidiosis and giardiasis through agricultural use of reclaimed wastewater. Int J Environ Health Res. 2006;16(6):405-18. doi: 10.1080/09603120601095100 PMID: 17164167.
15. Teodorovic S, Braverman JM, Elmendorf HG. Unusually low levels of genetic variation among *Giardia lamblia* isolates. Eukaryot Cell. 2007;6(8):1421-30. doi: 10.1128/EC.00138-07 PMID: 17557879.
16. Bonhomme J, Le Goff L, Lemée V, Gargala G, Ballet JJ, Favennec L. Limitations of tpi and bg genes sub-genotyping for characterization of human *Giardia duodenalis* isolates. Parasitol Int. 2011;60(3):327-30. doi: 10.1016/j.parint.2011.05.004 PMID: 21627998.
17. Kosuwin R, Putaporntip C, Pattanawong U, Jongwutiwes S. Clonal diversity in *Giardia duodenalis* isolates from Thailand: evidences for intragenic recombination and purifying selection at the beta giardin locus. Gene. 2010;449(1-2):1-8. doi: 10.1016/j.gene.2009.09.010 PMID: 19796671.
18. Alonso JL, Amorós I, Cuesta G. LNA probes in a real-time TaqMan PCR assay for genotyping of *Giardia duodenalis* in wastewaters. J Appl Microbiol. 2010;108(5):1594-601. doi: 10.1111/j.1365-2672.2009.04559.x PMID: 19840182.
19. Coronato Nunes B, Pavan MG, Jaeger LH, Monteiro KJ, Xavier SC, Monteiro FA, et al. Spatial and Molecular Epidemiology of *Giardia intestinalis* Deep in the Amazon, Brazil. PLoS One. 2016;11(7):e0158805. doi: 10.1371/journal.pone.0158805 PMID: 27392098.
20. Hussein AI, Tokoro M. Unpublished. Avaible in {HYPERLINK "https://www.ncbi.nlm.nih.gov/nuccore/AB480877"}.
21. Abe N, Teramoto I. Molecular evidence for person-to-person transmission of a novel subtype in *Giardia duodenalis* assemblage B at the rehabilitation institution for developmentally disabled people. Parasitol Res. 2012;110(2):1025-8. doi: 10.1007/s00436-011-2564-4 PMID: 21786066.
22. Tokoro M, Mizuno T, Hendarto J, Matey EJ, Songok EM, Ichimura H. Unpublished. Avaible in {HYPERLINK "https://www.ncbi.nlm.nih.gov/nuccore/LC508615"}.
23. Köster PC, Malheiros AF, Shaw JJ, Balasegaram S, Prendergast A, Lucaccioni H, et al. Multilocus Genotyping of *Giardia duodenalis* in Mostly Asymptomatic Indigenous People from the Tapirapé Tribe, Brazilian Amazon. *Pathogens*. 2021;10(2):206. https://doi.org/10.3390/pathogens10020206.
24. Paz e Silva FM, Monobe MM, Lopes RS, Araujo JP Jr. Molecular characterization of *Giardia duodenalis* in dogs from Brazil. Parasitol Res. 2012;110(1):325-34. doi: 10.1007/s00436-011-2492-3 PMID: 21695567.
25. Cacciò SM, De Giacomo M, Pozio E. Sequence analysis of the beta-giardin gene and development of a polymerase chain reaction-restriction fragment length polymorphism assay to genotype Giardia duodenalis cysts from human faecal samples. Int J Parasitol. 2002;32(8):1023-30. doi: 10.1016/s0020-7519(02)00068-1 PMID: 12076631.
26. Lalle M, Pozio E, Capelli G, Bruschi F, Crotti D, Cacciò SM. Genetic heterogeneity at the beta-giardin locus among human and animal isolates of *Giardia duodenalis* and identification of potentially zoonotic subgenotypes. Int J Parasitol. 2005;35(2):207-13. doi: 10.1016/j.ijpara.2004.10.022 PMID: 15710441.
